# The complete mitochondrial genome of *Mantis religiosa* (Mantodea: Mantidae) from Canada and its phylogeny

**DOI:** 10.1080/23802359.2019.1681912

**Published:** 2019-10-25

**Authors:** Yi-Yang Jia, Le-Ping Zhang, Xiao-Dong Xu, Xin-Yi Dai, Dan-Na Yu, Kenneth B. Storey, Jia-Yong Zhang

**Affiliations:** a College of Chemistry and Life Science, Zhejiang Normal University, Jinhua, Zhejiang Province, China;; b Key Lab of Wildlife Biotechnology, Conservation and Utilization of Zhejiang Province, Zhejiang Normal University, Jinhua, Zhejiang Province, China;; c Department of Biology, Carleton University, Ottawa, Canada

**Keywords:** Mantidae, mitogenome, phylogeny, *Mantis religiosa*

## Abstract

The complete mitochondrial genome of *Mantis religiosa* (Mantodea: Mantidae) from Canada was successfully sequenced. The mitochondrial genome was a circular molecule of 15,521 bp in length, containing 13 protein-coding genes, two rRNA genes, 23 tRNA genes (including an extra tRNA^Arg^ gene), and the control region. The AT content of the whole genome was 76.9% and the length of the control region was 636 bp with 81.9% AT content. The structure of the *M. religiosa* mitochondrial genome from Canada was almost identical to *M. religiosa* from China and their genetic distance was just 0.017. In Bayesian inference (BI) and maximum likelihood (ML) analyses, we found that *M. religiosa* was a sister clade to *Statilia* sp. and the monophyly of the genera Hierodula and Rhombodera was not supported.

*Mantis religiosa* (Mantodea: Mantidae), the European Mantis, is widely distributed across the world. The species occurs in China where it is characterized by an additional tRNA-Arg gene in the mitochondrial genome (Ye et al. 2016). It is listed as an introduced species in Canada (Kevan 1979; Miskelly and Paiero 2019). In this study, we sequenced the mitochondrial genome of *M. religiosa* from Canada to compare the gene structure with the Chinese counterpart and provide more molecular data to discuss the phylogenetic relationship within Mantidae.

The sample of *M. religiosa* was collected from Upper Canada Village (N 45°3′55″, E 74°56′34″), Morrisburg, Ontario, Canada. The sample (CA20170830-1) was identified and stored at −40 °C in the Animal Specimen Museum, College of Life Sciences and Chemistry, Zhejiang Normal University, China. Total genomic DNA was extracted from leg muscle using an Ezup Column Animal Genomic DNA Purification Kit (Sangon Biotech Company, Shanghai, China) and stored in the Zhang laboratory. A set of modified universal primers (Zhang et al. 2008; Zhang, Yu, et al. 2018) were designed for polymerase chain reaction (PCR) amplification. All PCR products were sequenced in both directions by the Sangon Biotech Company (Shanghai, China). The mitochondrial genome is deposited in GenBank with accession number MN356097.

The phylogenetic relationship was constructed using the Bayesian Inference (BI) (Ronquist et al. 2012) and RAxML 8.2.0 (Stamatakis 2014) softwares. To select conserved regions of the putative nucleotide sequences, each alignment was analyzed with Gblocks 0.91 b (Castresana 2000) using default settings. BI and ML trees were constructed using the 13 protein-coding genes of 21 species, which included 18 Mantidae species (Cameron et al. 2006; Song et al. 2016; Ye et al. 2016; Tian et al. 2017; Zhang and Ye 2017; Zhang, Yu et al. 2018; Zhang et al. 2018a, 2018b ) and three Blattaria species as the outgroups (Cameron and Whiting 2007; Dietrich and Brune 2016).

The complete mitogenome of *M. religiosa* from Canada was circular and 15,521 bp in length, containing the 38 mitochondrial genes including 13 protein-coding genes, 23 transfer RNA genes (including an additional tRNA^Arg^ gene), 2 ribosomal RNA genes. The same situation is typically found in the mitogenomes of other mantises (Ye et al. 2016; Zhang et al. 2019). The AT content of the complete mtDNA was 76.9% and the length of the control region was 636 bp with 81.9% AT content. Most protein-coding genes began with ATN (N represents A, T, C, G) as the start codons whereas the *CO1* gene began with TTG. *COX2* and *ND5* genes ended with an incomplete stop codon (T–) whereas the remaining 11 protein-coding genes ended with TAA. The structure of the mitochondrial genome of the specimen from Canada was very similar to *M. religiosa* from China and their genetic distance was 0.017. The phylogenetic relationships inferred from the BI and ML analyses shared almost similar topologies (Figure 1). *Mantis religiosa* was a sister clade to *Statilia* sp. and the monophyly of the genera *Hierodula* and *Rhombodera* were not supported, which agrees with the previous results of Zhang and Ye (2017) (Figure 1).

**Figure 1.**
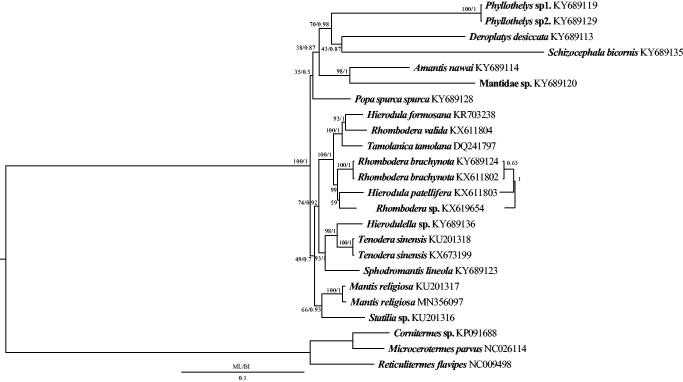
Phylogenetic tree of the relationships among 18 species of Mantodea based on the first and the second codon positions of the 13 mitochondrial protein-coding genes of 7338 nucleotides. Three termite species were included as the outgroups (*Cornitermes* sp., *M. parvus*, *R. flavipes*). Numbers around the nodes are the bootstrap values of ML on top and the posterior probabilities of BI on the bottom. The GenBank numbers of all species are shown in the figure.

## References

[CIT0001] Cameron SL, Barker SC, Whiting MF 2006 Mitochondrial genomics and the new insect order Mantophasmatodea. Mol Phylogenet Evol. 38:274–279. 16321547 10.1016/j.ympev.2005.09.020

[CIT0002] Cameron SL, Whiting MF 2007 Mitochondrial genomic comparisons of the subterranean termites from the Genus *Reticulitermes* (Insecta: Isoptera: Rhinotermitidae). Genome. 50:188–202. 17546084 10.1139/g06-148

[CIT0003] Castresana J 2000 Selection of conserved blocks from multiple alignments for their use in phylogenetic analysis. Mol Biol Evol. 17:540–552. 10742046 10.1093/oxfordjournals.molbev.a026334

[CIT0004] Dietrich C, Brune A 2016 The complete mitogenomes of six higher termite species reconstructed from metagenomic datasets (*Cornitermes* sp., *Cubitermes ugandensis*, *Microcerotermes parvus*, *Nasutitermes corniger*, *Neocapritermes taracua*, and *Termes hospes*). Mitochondrial DNA A DNA Mapp Seq Anal. 27:3903–3904. 25471441 10.3109/19401736.2014.987257

[CIT0005] Kevan D 1979 Dictuoptera. Vol. 111 In: Danks HV, editor. Canada and its insect fauna (Memoirs of the Entomological Society of Canada). Entomological Society of Canada; p. 314–316. Cambridge, England: Cambridge University Press.

[CIT0006] Miskelly J, Paiero SM 2019 Mantodea, Blattodea, Orthoptera, Dermaptera, and Phasmida of Canada. ZK. 819:255–269. 10.3897/zookeys.819.27241 PMC6355752 30713444

[CIT0007] Ronquist F, Teslenko M, Mark PVD, Ayres DL, Darling A, Höhna S, Larget B, Liu L, Suchard MA, Huelsenbeck JP 2012 MrBayes 3.2: efficient Bayesian phylogenetic inference and model choice across a large model space. Syst Biol. 61:539–542. 22357727 10.1093/sysbio/sys029 PMC3329765

[CIT0008] Song N, Li H, Song F, Cai W 2016 Molecular phylogeny of Polyneoptera (Insecta) inferred from expanded mitogenomic data. Sci Rep. 6:36175. 27782189 10.1038/srep36175 PMC5080581

[CIT0009] Stamatakis A 2014 RAxML version 8: a tool for phylogenetic analysis and post-analysis of large phylogenies. Bioinformatics. 30:1312–1313. 24451623 10.1093/bioinformatics/btu033 PMC3998144

[CIT0010] Tian XX, Liu J, Cui Y, Dong PZ, Zhu Y 2017 Mitochondrial genome of one kind of giant Asian mantis, *Hierodula formosana* (Mantodea: Mantidae). Mitochondrial DNA A. 28:11–12. 10.3109/19401736.2015.1106519 26641534

[CIT0011] Ye F, Lan X, Zhu WB, You P 2016 Mitochondrial genomes of praying mantises (Dictyoptera, Mantodea): rearrangement, duplication, and reassignment of tRNA genes. Sci Rep. 6:25634. 27157299 10.1038/srep25634 PMC4860592

[CIT0012] Zhang HL, Ye F 2017 Comparative mitogenomic analyses of praying mantises (Dictyoptera, Mantodea): origin and evolution of unusual intergenic gaps. Int J Biol Sci. 13:367. 28367101 10.7150/ijbs.17035 PMC5370444

[CIT0013] Zhang JY, Zhou CF, Gai YH, Song DX, Zhou KY 2008 The complete mitochondrial genome of *Parafronurus youi* (Insecta: Ephemeroptera) and phylogenetic position of the Ephemeroptera. Gene. 424:18–24. 18725275 10.1016/j.gene.2008.07.037

[CIT0014] Zhang LP, Cai YY, Yu DN, Storey KB, Zhang JY 2018a The complete mitochondrial genome of *Psychomantis borneensis* (Mantodea: Hymenopodidae). Mitochondrial DNA B. 3:42–43. 10.1080/23802359.2017.1419094 PMC7800046 33474058

[CIT0015] Zhang LP, Cai YY, Yu DN, Storey KB, Zhang JY 2018b Gene characteristics of the complete mitochondrial genomes of *Paratoxodera polyacantha* and *Toxodera hauseri* (Mantodea: Toxoderidae). PEERJ. 6:e4595. 29686943 10.7717/peerj.4595 PMC5911385

[CIT0016] Zhang LP, Ma Y, Yu DN, Storey KB, Zhang JY 2019 The mitochondrial genomes of *Statilia maculata* and *S. nemoralis* (Mantidae: Mantinae) with different duplications of trnR genes. Int J Biol Macromol. 121:839–845. 30340009 10.1016/j.ijbiomac.2018.10.038

[CIT0017] Zhang LP, Yu DN, Storey KB, Cheng HY, Zhang JY 2018 Higher tRNA gene duplication in mitogenomes of praying mantises (Dictyoptera, Mantodea) and the phylogeny within Mantodea. Int J Biol Macromol. 111:787–795. 29307803 10.1016/j.ijbiomac.2018.01.016

